# Editorial of Special Issue “Non-Coding RNAs in Pathogen-Host Interaction”

**DOI:** 10.3390/ijms222111346

**Published:** 2021-10-21

**Authors:** Yong Sun Lee

**Affiliations:** Department of Cancer Biomedical Science, Graduate School of Cancer Science and Policy, National Cancer Center, Goyang 10408, Korea; yslee@ncc.re.kr; Tel.: +82-31-920-2748; Fax: +82-31-920-2759

Clinical outcomes after pathologic infection are variable in infected individuals, ranging from no symptoms, moderate symptoms, hospitalization, and even death. This outcome diversity is attributed not only to the virus itself but also largely to each person’s genetic/epigenetic characteristics. Inter-personal genomic/epigenomic variations and the resulting differences in gene expression profiles determine the process of infection as well as its clinical manifestation. Therefore, understanding the complexity of host–pathogen interactions and their effect on gene expression is imperative to develop treatments and prevent future infectious diseases.

Over the last two decades, non-coding RNAs (ncRNAs) have emerged as important regulators of gene expression and the resulting phenome. This new knowledge has shifted our conventional concept of classic ncRNAs (such as tRNAs and rRNAs) that are constitutively expressed and play an invariable role in translation, which is a fundamental biological process. Fueled by the development of next-generation sequencing technology, a sensitive and high-throughput tool that captures a myriad of transcripts, researchers are now intensely studying various types of regulatory ncRNAs.

At the crossroad of infectious diseases and regulatory ncRNAs, we arranged this Special Issue entitled “Non-coding RNAs in Pathogen-Host Interaction” and have compiled eight fascinating papers that cover clinically important pathogens and scientifically interesting ncRNAs. A brief outlook of these individual papers follows below.

Pathogens covered in this Special Issue are mostly viruses, although a review paper by Morishita et al. [1] has also discussed bacterial infections and liver parasites. A diverse range of viruses are discussed in this Special Issue, including enteroviruses, respiratory syncytial virus (RSV), Epstein–Barr virus (EBV), Rift valley fever virus, Japanese encephalitis virus, Zika virus, Hepatitis B virus (HBV), and Hepatitis C virus (HCV), all important pathogens. Enteroviruses are a genus of small RNA viruses that cause various types of human diseases (listed in [2]) and are well associated with ncRNAs. These findings are summarized in a review paper by Zhu et al. [2]. RSV infects the lower respiratory tract and can be fatal in infants, older adults, and immunocompromised individuals. Here, Choi et al. [3] have reported the mechanism of action of a small regulatory ncRNA derived from tRNA during RSV infection. EBV, in addition to its established role in cancer, has been implicated in a variety of other diseases. One of them is multiple sclerosis (MS), in which Afrasiabi et al. [4] investigated a potential role of host and EBV-encoded miRNAs. Rift valley fever virus, Japanese encephalitis virus, and Zika virus are all deadly viruses. Two elegant works from Patel laboratory [5,6] characterized molecular interactions between host proteins (DDX17 and DDX3X) and viral RNAs by in-depth biophysical analysis.

miRNAs and long ncRNAs are the two main groups of regulatory ncRNAs. In this Special Issue, Morishita et al. summarize the role of miRNAs in hepatic immunology [1] while Afrasiabi et al. analyzed miRNAs in relation to the risk of single nucleotide polymorphism in MS patients [4]. In addition to these articles, Elsayed et al. [7] report on the role of multiple long ncRNAs, one in particular “PRKAR1B-AS2”, that is shown to play a prominent role in ovarian cancer. In addition to miRNAs and long ncRNAs, two other types of ncRNAs are presented in this Special Issue. One is tRNA-derived RNA Fragments (tRFs), which are an emerging class of small ncRNAs with diverse biological functions. Choi et al. [3] characterized the molecular function of an RSV-induced tRF. The other is an ncRNA called “nc886”, which is beginning to gain attention as it possesses distinct features different from other regulatory ncRNAs. Lee et al. [8] has reported on nc886′s role in the interferon response, a primary host defense mechanism against pathogen intrusion.

The works presented within this Special Issue represent endeavors to understand the cross-talk between ncRNAs and pathogen infection. As our knowledge about their interaction expands and deepens, we will move one step closer to subduing infectious diseases and potential future pandemics.
